# Development of a new cucumber reference material for pesticide residue analysis: feasibility study for material processing, homogeneity and stability assessment

**DOI:** 10.1007/s00216-015-8476-x

**Published:** 2015-01-28

**Authors:** Susana Grimalt, Stefan Harbeck, Penka Shegunova, John Seghers, Berit Sejerøe-Olsen, Håkan Emteborg, Marta Dabrio

**Affiliations:** European Commission, Joint Research Centre, Institute for Reference Materials and Measurements (IRMM), 111 Retieseweg, 2440 Geel, Belgium

**Keywords:** Food safety, Certified reference material, Pesticide residue analysis, Homogeneity study, Stability study

## Abstract

The feasibility of the production of a reference material for pesticide residue analysis in a cucumber matrix was investigated. Cucumber was spiked at 0.075 mg/kg with each of the 15 selected pesticides (acetamiprid, azoxystrobin, carbendazim, chlorpyrifos, cypermethrin, diazinon, (α + β)-endosulfan, fenitrothion, imazalil, imidacloprid, iprodione, malathion, methomyl, tebuconazole and thiabendazole) respectively. Three different strategies were considered for processing the material, based on the physicochemical properties of the vegetable and the target pesticides. As a result, a frozen spiked slurry of fresh cucumber, a spiked freeze-dried cucumber powder and a freeze-dried cucumber powder spiked by spraying the powder were studied. The effects of processing and aspects related to the reconstitution of the material were evaluated by monitoring the pesticide levels in the three materials. Two separate analytical methods based on LC–MS/MS and GC–MS/MS were developed and validated in-house. The spiked freeze-dried cucumber powder was selected as the most feasible material and more exhaustive studies on homogeneity and stability of the pesticide residues in the matrix were carried out. The results suggested that the between-unit homogeneity was satisfactory with a sample intake of dried material as low as 0.1 g. A 9-week isochronous stability study was undertaken at −20 °C, 4 °C and 18 °C, with −70 °C designated as the reference temperature. The pesticides tested exhibited adequate stability at −20 °C during the 9-week period as well as at −70 °C for a period of 18 months. These results constitute a good basis for the development of a new candidate reference material for selected pesticides in a cucumber matrix.

**Electronic supplementary material:**

The online version of this article (doi:10.1007/s00216-015-8476-x) contains supplementary material, which is available to authorized users.

## Introduction

The state of the art of analytical instruments currently applied to the analysis of contaminants in food has enabled the production of highly accurate data even at low concentration levels. In the case of pesticide residue analysis, a large number of compounds are usually simultaneously detected by means of MS using multi-residue methods (MRM). The wide range of different chemical structures present among pesticides can compromise the accuracy of the MRM for certain analytes. Additionally, the limited availability of official methods of analysis, covering every matrix and pesticide combination on the market, usually requires the in-house development and validation of methods specific for the laboratories’ own purposes. To ensure the reliability and comparability of the analytical results, laboratories are expected to have appropriate quality assurance systems in place. The International Organization for Standardization (ISO) describes in the ISO/IEC 17025 standard [1] the general requirements for the quality assurance of test results including the regular use of certified reference materials (CRMs). Thus, one essential tool to assess the quality of the measurement results is the use of CRMs. A CRM can be used during method validation to assess trueness and ensure traceability of the results. Furthermore, the guidance document on analytical quality control and validation procedures for pesticide residues analysis in food and feed published by the European Commission refers to CRMs as the preferred option to provide evidence of the method’s performance [2]. Once the method is validated, a CRM can be included in the analysis of routine samples as a quality control tool in order to verify the performance of the analytical method over time.

Although several CRMs for organochlorine pesticides (OCPs) and polychlorinated biphenyls (PCBs) have been developed, they are mainly related to environmental matrices [3]. In the food sector only a limited number of pesticides (fenitrothion, diazinon, cypermethrin and chlorpyrifos) have been recently certified in vegetable or fruit matrices, such as brown rice (NMIJ-CRM 7504-a) [4], green onion (NMIJ-CRM 7507-a), cabbage (NMIJ-CRM 7508-a) [5], soybean (NMIJ-CRM 7509-a) [6] and apple (NMIJ-CRM 7510-a) [7]. Besides these materials, there are still a vast number of pesticide families and vegetable/fruit–matrix/combinations that are not covered by any commercial matrix CRM. One of the main difficulties is the tendency of some pesticides to degrade in natural matrices. Therefore the stabilisation of the pesticides in the matrix becomes critical within the reference material production process. Prior to the production of a new CRM in this field, several considerations have to be taken into account [8]. Basically the following steps are required:

- Devise a strategy for the selection of the analyte–matrix combination including representative pesticides from different chemical compound families [9]; a matrix representing one of the commodity groups as classified by European guidelines [2]; a successful combination inside the scope of the legal priorities [10].
- Design and perform a feasibility study on the analyte–matrix behaviour depending on the preparation, processing and storage conditions. Ideally a CRM aims to mimic the properties of real samples as close as possible. Therefore, minimal manipulation of the matrix and the best conservation of the analytes of interest are desirable. Aspects related to homogeneity and stability [11] of the target analytes in the candidate material are investigated to determine the suitability of the material to become a CRM.
- Development of suitable analytical methodology for monitoring the analytes in the selected matrix during the feasibility study and applicable for the eventual characterization of the candidate CRM. The latter role requires high accuracy and a suitable level of measurement uncertainty. Two crucial factors are kept in mind to obtain highly accurate measurements: the use of pure and stable calibrants, and the application of validated methods. Full method validation is conducted in compliance with ISO/IEC 17025 according to stringent validation criteria.
- If successful, the feasibility study may continue with the preparation of a candidate CRM. The candidate CRM is processed, investigated for homogeneity and stability, and followed by a characterization campaign usually organised as an interlaboratory exercise [12].

The lack of matrix reference materials (RMs) for pesticides in vegetables with a high content of water makes cucumber, as a representative commodity of this group, a good target matrix for a candidate CRM. This paper reports the findings achieved during the feasibility studies for the production of an RM for 15 pesticides each at a target level of 0.075 mg/kg in fresh cucumber. Three different processing strategies were evaluated in order to find the optimal conditions for minimal material alteration and pesticide degradation. Homogeneity and short-term stability studies of the processed spiked cucumber were performed. The analysis of the samples required the in-house development of two analytical methods, presented here, which were fully validated respecting the method performance parameters specified in ISO/IEC 17025.

## Experimental

### Analytical methodology

Two complementary analytical methods were developed and validated at the Institute for Reference Materials and Measurements (IRMM) for use in the study of the cucumber material. One is based on liquid chromatography (LC) and the other on gas chromatography (GC), both coupled to tandem mass spectrometry (MS/MS) for the analysis of acetamiprid, azoxystrobin, carbendazim, chlorpyrifos, cypermethrin, diazinon, α- and β-endosulfan, fenitrothion, imazalil, imidacloprid, iprodione, malathion, malaoxon, methomyl, tebuconazole and thiabendazole. A stable isotopically labelled standard solution containing acetamiprid-*d*_3_, azoxystrobin-*d*_4_, carbendazim-*d*_4_, chlorpyrifos-*d*_10_, cypermethrin-^13^ *C*_6_, diazinon-*d*_10_, β-endosulfan-^13^ *C*_9_, fenitrothion-*d*_6_, imazalil-*d*_5_, imidacloprid-*d*_4_, iprodione-*d*_7_, malathion-*d*_10_, malaoxon-*d*_6_, methomyl-*d*_3_, tebuconazole-*d*_6_ and thiabendazole-*d*_6_ was always added to aid quantification.

#### Chemicals

The pesticide standards used for analytical purposes were purchased from Dr. Ehrenstorfer (Augsburg, Germany), CDN Isotopes (Quebec, Canada), Cambridge Isotope Laboratories INC. (MA, USA) and High Purity Compounds (Cunnersdorf, Germany) with purities higher than 97 %, except for 94 % in the case of malathion. The pesticides for spiking the material during processing were divided into two types: pure solid standards grade Pestanal (95.2–99.9 % purity) from Sigma Aldrich (Bornem, Belgium) such as azoxystrobin, carbendazim, diazinon, endosulfan, fenitrothion, imazalil, malathion, methomyl and thiabendazole; and, commercial formulations such as acetamiprid (Kb Multisect concentration 5 g/L; Scotts Miracle-Gro CO, Marysville, Ohio), chlorpyrifos (Pychlorex GR concentration 5 %; Edialux, Bornem Belgium), cypermethrin (Aveve concentration 10 g/L; Leuven, Belgium), imidacloprid (Provado Ultra concentration 10 g/L; Bayer, Levekursen Germany), iprodione (Rovral SC concentration 500 g/L; BASF, Ludwigshafen Germany) and tebuconazole (Rosacur concentration 43 g/L, Bayer). Acetone, acetonitrile, ethyl acetate, cyclohexane, methanol and toluene LC–MS hypergrade or pesticide grade solvents were acquired from Merck (Darmstadt, Germany). Water UPLC-grade, ammonium acetate for MS, sodium sulphate pure grade, solid phase extraction (SPE) cartridges Supleco Discovery DSC18 SPE and PSA SPE 500 mg (6 mL) were purchased from Sigma-Aldrich. Ultrapure water from a MilliQ water purification system from Millipore (Brussels, Belgium) was used.

#### Instrumentation

##### LC–MS/MS

The analytes covered by this method were azoxystrobin, acetamiprid, carbendazim, chlorpyrifos, diazinon, imazalil, imidacloprid, malathion, malaoxon, methomyl, thiabendazole and tebuconazole. An ultra-performance liquid chromatography (UPLC) Acquity Waters system was interfaced to a Xevo TQ-S triple quadrupole mass spectrometer (Waters, Milford, MA, USA) using an electrospray interface (ESI). The UPLC separation was performed by injecting 2.5 μL of sample extract onto an Acquity UPLC BEH C_18_ (2.1 × 100 mm i.d., 1.7 μm) (Waters) column. The mobile phase used was a water/methanol gradient with 1 mM ammonium acetate where the percentage of methanol was linearly changed as follows: 0.0 min, 5 %; 1.0 min, 5 %; 6.0 min, 95 %; 7.5 min, 95 %; 7.8 min, 5 %; 10.0 min, 5 %, which was maintained at a flow rate of 400 μL/min. Nitrogen was used as the desolvation gas and cone gas at flow rates of 1,000 and 150 L/h, respectively. For MS/MS mode operation, the collision gas was argon 99.9 % from Praxair (Schoten, Belgium) at a flow rate of 0.18 mL/min. A capillary voltage of 3.00 kV and source offset of 60 V were used in the positive ionization mode. The desolvation temperature was set to 350 °C. Three MS/MS transitions were selected for each analyte, one selected reaction monitoring (SRM) transition was used for quantification and the other two for confirmation purposes (see Electronic Supplementary Material (ESM), Annex I). The programme TargetLynx was used to process the quantitative data.

##### GC–MS/MS

The analytes covered by this method included cypermethrin, α- and β-endosulfan, fenitrothion and iprodione. A Trace 1310 GC oven was coupled to a TSQ Quantum XLS Ultra triple quadrupole (Thermo Scientific Interscience (Waltham, MA, USA)) with electron ionisation. The GC separation was performed by injecting 1 μL of sample extract onto a GC column TG-SQC (20 m × 0.25 mm i.d. × 0.25 μm film) by a split/splitless injector in splitless mode with purge at 500 kPa (purge time 1 min). Helium 99.9 % pure (Praxair) as a carrier gas was used at a constant flow of 1.5 mL/min. The temperature gradient was initiated at 120 °C, where it was held for 1.0 min, increased at 15 °C/min until 300 °C, and maintained at 300 °C for 4 min. The temperature of the transfer line was kept constant at 300 °C. For the MS/MS mode operation, argon 99.9 % (Praxair) was used as the collision gas at a pressure of 1.5 mTorr. Three SRM transitions were selected for each analyte, one used for quantification and two for confirmation purposes (see ESM, Annex II). The XCalibur software was applied to process the data.

#### Analytical procedure

##### Sample extraction for LC–MS/MS analysis

Two grams of fresh or reconstituted cucumber sample were weighed into a 10 mL PTFE tube and spiked with the surrogate mixture solution to a 1 ng/mL level in the sample extract (0.01 mg/kg in the sample). A methanol/water solvent mixture (75:25 v/v, 10 mL) was added and then placed in an automatic shaking system (KS501digital, IKA Labortechnik, Staufen, Germany) during 60 min at 300 rpm. The extract was filtered through a folded cellulose filter and made up to a volume of 20 mL. An aliquot was filtered through Nylon 0.2 μm directly into a 1.5-mL vial and injected onto the LC–MS/MS system.

##### Sample extraction for GC–MS/MS analysis

Five grams of cucumber sample (200 mg of freeze-dried sample reconstituted by adding 5 g H_2_O) was weighed into a 50-mL PTFE tube, and spiked with the surrogate mixture solution equivalent to 0.03 mg/kg wet sample. Ethyl acetate (20 mL) was added and then shaken for 20 min at 300 rpm (KS501 digital, IKA Labotechnik, Staufen, DE). The sample extract was centrifuged (Multifuge X1R, Hereus, DE) for 5 min at 4,000 rpm, and the liquid phase was transferred to a round-bottom flask. This extraction was repeated and the two aliquots were combined in the round flask, to which 5 ml of acetonitrile was added and evaporated on a rotatory evaporator (Laborota 4001, Heidolph, DE) to approximately 1 mL. Additionally 5 mL of acetonitrile was added and evaporated prior to transferring the flasks’ contents into an automated SPE system (Gx-274 Aspect, Gilson, USA). A two-step SPE procedure was followed using C_18_ and PSA cartridges, respectively, both preconditioned with 5 mL acetonitrile. The elution from the C_18_ SPE cartridge was done using 5 mL of acetonitrile, and then the collected extract was evaporated under a stream of nitrogen to 1 mL. This extract was loaded onto the PSA SPE cartridge and eluted with 4 mL of acetonitrile, and the collected extract was evaporated under a stream of nitrogen to near dryness. Approximately 200 μL of toluene was added to the extract and transferred to a vial, with a low volume micro-insert and finally injected onto the GC–MS/MS system.

Both analytical procedures were validated and it was shown that they sufficiently fulfilled the performance criteria established by the European guidelines [2] with an associated relative expanded uncertainty lower than 10 % (Table 1).

**Table 1:** General information on the selected pesticides and the analytical procedures based on LC–MS/MS and GC–MS/MS

Analyte	Functionality/chemical group	MRL (mg/kg)	LOQ (mg/kg) × 10^−3^	*s*_rel rep_ (%)	*U*_rel_ (%)
Acetamiprid^a^	Insecticide/neonicotinoid	0.3	0.031	1.8	2.9
Azoxystrobin^a^	Fungicide/strobilurin	1	0.063	1.8	5.1
Carbendazim^a^	Fungicide/carbamate	0.1	0.40	4.4	10.7
Chlorpyrifos^a^	Insecticide/organothiophosphate	0.05	0.067	1.6	6.4
Cypermethrin^b^	Insecticide/pyrethroid	0.2	2.4	1.3	8.4
Diazinon^a^	Insecticide/organothiophosphate	0.01	0.057	1.8	4.9
α + β Endosulfan^b,c^	Insecticide/organochlorine	0.05	6.2	4.2	14.0
Fenitrothion^b^	Insecticide/organothiophosphate	0.01	1.7	1.6	6.6
Imazalil^a^	Fungicide/conazole	0.2	0.061	2.6	19.0
Imidacloprid^a^	Insecticide/neonicotinoid	1	0.17	1.9	5.4
Iprodione^b^	Fungicide/imidazole	2	0.9	6.5	12.1
Malathion + malaoxon^a,d^	Insecticide/organothiophosphate	0.02	0.029	2.2	3.5
Methomyl^a^	Insecticide/oxime carbamate	0.02	0.028	1.8	4.2
Tebuconazole^a^	Fungicide/conazole	0.5	0.087	1.6	7.1
Thiabendazole^a^	Fungicide/thiazole benzimidazole	0.05	0.049	3.1	9.5

*MRL* maximum residue level, *LOQ* limit of quantification calculated as signal to noise equal to 10, *U*_*rel*_ relative expanded uncertainty of the procedure, for 3 replicates analysed in 1 day (*k* = 2)

^a^Analysed by LC–MS/MS

^b^Analysed by GC–MS/MS

^c^Validation done considering the addition of the peaks of the two isomers α-endosulfan and β-endosulfan

^d^Validation carried out considering the residue definition as sum of malathion and malaoxon expressed as malathion

### Design of the feasibility study

To establish the best conditions for the preparation of a CRM, three basic steps should be considered: (a) study of different processing strategies for minimizing changes of the analyte(s) and the matrix during preparation, handling and storage of the material; (b) study of the homogeneity of the material at a given representative sample intake; and, (c) study of the stability of the material during storage and transport.

#### Preparation and processing of the material

##### Preparation of the spiked cucumber

The original material (*Cucumis sativus*), free of pesticides, was obtained from a local grocery store specialising in organically grown vegetables. The pesticides used for spiking the material were either high purity neat crystalline compounds (azoxystrobin, carbendazim, diazinon, endosulfan, fenitrothion, imazalil, malathion, methomyl and thiabendazole) from Sigma-Aldrich, or commercial pesticide preparations (acetamiprid, chlorpyrifos, cypermethrin, imidacloprid, iprodione and tebuconazole) as described above. An individual solution of each pesticide in methanol or water, depending on the solubility of the active compound, was prepared gravimetrically and mixed in water to obtain an approximate mass fraction of 0.075 mg/kg per pesticide in the cucumber slurry.

The fresh cucumber was cut into small cubes with a knife and milled at temperatures between −180 and −100 °C using a cryogenic mill (Humboldt-Wedag, Köln, DE). The milled cucumber was allowed to thaw and then mixed with a Silverson blender (Silverson, Chesham, UK) for 10 min to afford a highly homogenised slurry. A small portion of the cucumber slurry was taken aside to be mixed manually for 2 min with the pesticide solution. This mixture was added to the remaining cucumber slurry and mixed again for 5 min with the Silverson blender to create an homogeneous slurry. Part of the spiked cucumber material was aliquoted into 10-g portions by pouring into 65-mL glass jars and stored at −70 or −20 °C. The samples stored at −70 °C were used as a reference during the feasibility study to check for possible losses or degradation of the material during freeze-drying. Two different freeze-dried materials (A and B) were prepared in order to study the influence of processing conditions on the behaviour and stability of the pesticides.

##### Freeze-dried material A

The spiked slurry was frozen and freeze-dried in an Epsilon 2-100D freeze-dryer (Martin Christ, Osterode, DE), sieved through a 500-μm stainless steel mesh, and re-milled with the cryogenic mill. Then 50-mL amber glass vials were filled with the obtained freeze-dried powder (3 g) and stored in the dark at −70 °C.

##### Freeze-dried material B

An alternative strategy was checked by freeze-drying a portion of the blank cucumber slurry, which was subsequently sprayed with a pesticide solution. The same freeze-drying process as for material A was used. The blank freeze-dried powder obtained was spread evenly on a Teflon-coated tray and a mixture of pesticides in solution was sprayed using a Meinhard nebuliser (Meinhard, Golden CO, USA) to obtain a mass fraction of 0.075 mg/kg per pesticide in the reconstituted cucumber material. Thereafter a second vacuum drying was applied, followed by crushing and milling using a Fritsch-Pulverisette 14 (Fritsch, Ider-Oberstein, DE) mill with a gentle flow of liquid nitrogen. The final homogenization was done with a Dynamix CM200 mixer (WAB, Basel, Switzerland). Then 50-mL amber glass vials were filled with this material (3 g) and stored at −70 °C.

The water content of the freeze-dried material was measured by volumetric Karl Fischer titration (V-KFT) on two replicates of five randomly chosen vials [13]. Additionally, the particle size of the freeze-dried material was measured on five replicates of two randomly chosen vials by laser diffraction. Samples were analysed over a range of 0.5–1,750 μm using a Helos laser light scattering instrument (Sympatec GmbH System-Partikel-Technik, Clausthal-Zellerfeld, DE).

#### Homogeneity study

A key requirement for any RM is the equivalence between the various units. In this respect, it is relevant whether the variation between units is significant compared to the uncertainty of the certified value (ISO guide 34 [14]). Two sets of six units of material type A were chosen using a random stratified sample selection scheme, and three subsamples from each unit were analysed for their pesticide content with the in-house validated methods described earlier. One set of six units was analysed by LC–MS/MS and the other set by GC–MS/MS. The measurements were in both cases performed under repeatability conditions and in a randomised manner to be able to separate a potential analytical drift from a trend in the filling sequence. The resulting data were investigated for outliers using the Grubbs test, and linear regression analysis was performed to check for possible trends in the filling sequence. The distribution of results was checked using normal probability plots and histograms. Finally, analysis of variance (ANOVA) was performed to quantify the within-bottle standard deviation (*s*_wb_) and the between-bottle standard deviation (*s*_bb_). Moreover, the uncertainty due to possible inhomogeneity that can be hidden by the method repeatability (*u* ^*^_bb_) was calculated as described elsewhere [11].

The within-unit homogeneity is closely correlated with the minimum sample intake. The minimum sample intake is the minimum amount of sample that was representative of the whole unit. Sample sizes equal to or above the minimum sample intake guarantee the validity of the certified value within its stated uncertainty. To ensure that the amount of sample employed for analysis during the feasibility study was adequate and representative, an experiment was performed by LC–MS/MS selecting randomly a set of six units of material A. Aliquots of 50, 100 and 200 mg were tested in duplicate in each unit. The same statistical criteria adopted for the homogeneity study were considered in this study.

#### Stability study description

An isochronous stability study [15] was conducted during a period of 9 weeks to obtain information about the potentially optimal storage temperature for the candidate RM. Sets of two units for each analytical method were selected using a random stratified sampling scheme. They were stored at −20, 4 and 18 °C for 0, 3, 6 and 9 weeks at each temperature, with −70 °C acting as the reference temperature. For each unit, two independent replicates were analysed by LC–MS/MS and GC–MS/MS under repeatability conditions. An individual evaluation of the results for each temperature was undertaken. The data were checked for outliers using the Grubbs test, and a linear regression analysis as a function of time was performed. Slopes were tested for significance using a *t* test [16].

## Results and discussions

### Processing of the material

Two freeze-dried materials (type A, spiked freeze-dried and type B, blank freeze-dried and spiked by spraying) were studied to select the best processing workflow for pesticide spiking in the cucumber matrix. The influence of the different processing strategies on the pesticide mass fractions was investigated. For each processed material the pesticide recoveries were obtained considering the mass fraction of each pesticide in the solvent mixture prepared by weighing as the reference for each spiked material (Table 2). The type A material exhibited a high degree of similarity to the spiked fresh slurry for most of the analytes measured by LC–MS/MS, with the exception of a small loss of chlorpyrifos and diazinon. The comparison of the type B material and the slurry also showed a high degree of similarity, but with a slight tendency for better recovery values, especially for malathion, methomyl and thiabendazole, while for tebuconazole a loss of the analyte was observed during the spraying process, probably as a result of volatility or degradation by oxidation.

**Table 2:** Mean of recovery and RSD (*n* = 3) values for each analyte studied by LC–MS/MS in the three different processed materials

Analyte	Recovery (%) (RSD, %)		
	Spiked slurry	Freeze-dried material A	Freeze-dried material B
Acetamiprid	85 (4.2)	87 (1.2)	71 (2.3)
Azoxystrobin	96 (3.9)	97 (0.5)	98 (0.9)
Carbendazim	55 (7.1)	54 (4.1)	64 (6.8)
Chlorpyrifos	83 (5.2)	61 (2.4)	–^a^
Diazinon	74 (5.2)	48 (0.3)	73 (3.2)
Imazalil	59 (7.9)	48 (0.9)	61 (5.5)
Imidacloprid	77 (4.8)	77 (0.6)	61 (5.4)
Malathion + malaoxon	67 (4.8)	63 (1.7)	118 (6.3)
Methomyl	79 (4.1)	77 (1.5)	92 (1.0)
Tebuconazole	68 (6.1)	70 (1.3)	52 (20.0)
Thiabendazole	73 (4.4)	73 (1.1)	85 (1.2)

Recovery values are referred to the pesticide mass fractions in the spiking solution employed for material preparation

^a^Chlorpyrifos was not included in the spraying material B as a result of difficulties with the solubility of the agrichemical formulation

Regarding the use of pure standard vs commercial agrochemical of the active compounds for spiking, no clear analytical effect was linked to the use of one or the other; however, solubility issues were in some cases experienced, probably caused by excipients present in the commercial agrochemical mixtures.

The water content of freeze-dried material A, as determined by V-KFT of five units, was 6.02 ± 0.89 g/100 g; the freeze-drying resulted in a mass loss of about 96 % of the starting material (average result from the weighing of 12 freeze-drying trays). The cumulative particle size distribution for the freeze-dried material obtained by laser diffraction yielded about 920 μm for the 99th percentile as the top particle size.

### Homogeneity

The between-bottle homogeneity was evaluated in the two freeze-dried materials (A and B) as well as in the spiked fresh slurry. The data sets were tested for consistency using a Grubbs’ outlier test at a confidence level of 99 % on the individual results and the unit means. No outlying individual results were found for each pesticide in the three materials. Regression analysis indicated no statistically significant trend in the filling sequence of any compound, but a trend was found in the analytical sequence for diazinon and methomyl. Individual data and bottle means showed normal or at least unimodal distribution in all cases. Therefore, the uncertainty contribution from possible heterogeneity could be estimated by ANOVA [11]. Results are summarized in Table 3. Contributions (*s*_bb_ or *u* ^*^_bb_) of less than 4 % for all the analytes in the spiked slurry and material A indicated an acceptable level of homogeneity in both materials. Exceptionally, in the case of methomyl, a higher value for *s*_bb_ was obtained; however, this between-bottle standard deviation is inside the suitable range of homogeneity. Material B showed values for *s*_bb_ of higher than or equal to 7 %, but always lower than 10 % considered as acceptable. The data analysis indicated improved homogeneity characteristics for material A in comparison to material B, suggesting that material A is the better candidate for a potential CRM production.

**Table 3:** Homogeneity study: uncertainty estimations for frozen, freeze-dried materials A and B

Analyte	Frozen material			Freeze-dried material A			Freeze-dried material B		
	*s*_wb_ (%)	*s*_bb_ (%)	*u* ^*^_bb_ (%)	*s*_wb_ (%)	*s*_bb_ (%)	*u* ^*^_bb_ (%)	*s*_wb_ (%)	*s*_bb_ (%)	*u* ^*^_bb_ (%)
Acetamiprid	2.9	NC	**1.6**	1.1	**1.5**	0.4	1.5	**8.5**	0.8
Azoxystrobin	2.6	NC	**1.4**	5.4	**0.8**	2.0	1.9	**9.7**	1.0
Carbendazim	6.7	NC	**3.6**	3.9	**2.7**	1.4	1.8	**8.7**	1.0
Chlorpyrifos	2.7	**1.9**	1.4	1.6	**1.1**	0.3	–^a^	–^a^	–^a^
Cypermethrin	4.3	1.9	**2.5**	1.5	NC	**0.5**	3.9	**35.3**	2.5
Diazinon	2.2	NC	**1.2**	0.9	**1.8**	0.3	2.0	**8.7**	1.1
α + β Endosulfan	7.9	3.1	**4.6**	3.3	1.0	**1.2**	5.5	**51.5**	3.5
Fenitrothion	2.5	1.0	**1.5**	2.3	**1.3**	0.9	4.8	**33.7**	3.1
Imazalil	3.3	NC	**1.8**	1.5	**2.0**	0.5	3.9	**9.1**	2.1
Imidacloprid	2.9	NC	**1.5**	1.1	**1.5**	0.4	1.3	**8.7**	0.7
Iprodione	2.6	0.6	**1.5**	6.0	**4.1**	2.2	4.6	**29.3**	2.9
Malathion + malaoxon	2.5	**1.7**	1.3	2.0	**1.7**	0.7	4.3	**6.9**	2.3
Methomyl	1.8	**7.4**	1.0	3.0	**6.5**	1.1	3.8	**8.6**	2.0
Tebuconazole	3.4	NC	**1.8**	1.5	**1.1**	0.5	1.1	**9.6**	0.6
Thiabendazole	2.4	NC	**1.3**	1.8	**1.5**	0.6	2.2	**9.6**	1.2

Relevant homogeneity data for the given pesticide and treatment are in bold

*NC* cannot be calculated as MS_between_<MS_within_, *s*_*wb*_ within-bottle standard deviation, *s*_*bb*_ between-bottle standard deviation, u*_bb_ uncertainty due to possible inhomogeneity that can be hidden by the method repeatability

^a^Chlorpyrifos was not included in the experiment for freeze-dried material B because of solubility problems

The within-bottle homogeneity and minimum sample intake were assessed in the freeze-dried material A, which was stored at −70 °C. A quantitative analytical study of the sample intake was performed by LC–MS/MS on three sets of six samples each. Sample intakes of 50, 100 and 200 mg of powdered material A were tested. A mean value of each mass fraction level was calculated with its standard deviation, the latter shown in Table 4. Additionally, the standard deviation of the combined data pool for the three sample intakes was estimated and a Grubbs’ test for outlier screening for the three sets was carried out (Table 4). The *s*_bb_ values obtained were lower than 3.5 % for all analytes with the exception of carbendazim. The latter higher value of *s*_bb_ was caused by one outlier within the 50-mg sample intake set by a single Grubbs’ test that can also be observed by the increased value of the standard deviation for this individual set of six samples. During method validation an inferior method repeatability was obtained for carbendazim in comparison to other compounds. For conservative reasons and to minimise the influence of inhomogeneity, a minimum sample intake of 100 mg was recommended for freeze-dried material A.

**Table 4:** Mass fraction values for sample intakes of 50, 100 and 200 mg for the LC–MS/MS method

Sample intake	Mass fraction in dry material (mg/kg) (RSD, %)			*s*_bb_(%)	Outlying results
	50 mg	100 mg	200 mg		
Acetamiprid	1.51 (3)	1.51 (4)	1.50 (2)	2.6	None
Azoxystrobin	1.89 (2)	1.93 (1)	1.86 (1)	2.2	None
Carbendazim	0.73 (11)	0.68 (5)	0.75 (5)	8.6	Yes
Chlorpyrifos	1.18 (2)	1.21 (2)	1.19 (2)	1.9	None
Diazinon	1.05 (2)	1.08 (3)	1.09 (2)	2.9	None
Imazalil	0.89 (3)	0.90 (4)	0.90 (3)	3.5	None
Imidacloprid	1.71 (2)	1.72 (2)	1.70 (1)	2.0	None
Malathion + malaoxon	1.42 (3)	1.40 (2)	1.40 (2)	2.3	None
Methomyl	2.29 (1)	2.35 (2)	2.45 (2)	3.2	None
Tebuconazole	1.00 (1)	0.99 (3)	0.97 (3)	2.4	None
Thiabendazole	1.31 (3)	1.31 (3)	1.29 (2)	2.6	None

Outliers from the total set of values were screened by single and double Grubbs’ test with a confidence level of 99 %

*s*_*bb*_ deviation value for the data pool of the three sets of measurements

### Stability

The stability study was carried out on the freeze-dried material A at three temperatures/four time intervals following an isochronous scheme. The obtained data were evaluated individually for each temperature. The results were screened for outliers using the single and double Grubbs’ test with a confidence level of 99 %. No outlying individual results were found in the data for any temperature during the isochronous study. Furthermore, the data were evaluated against storage time by regression analysis of response vs. time of storage. The slopes of the regression functions were tested for statistical significance, as indicated in Table 5. Statistically the slopes were not significantly different from zero at −20 °C for most of the analytes. As an exception, α + β endosulfan and fenitrothion showed a negative trend that was significant, likely as a consequence of limited stability at −20 °C. In the case of azoxystrobin at −20 °C, a positive trend was observed, although the difference between the highest and lowest values remained within the uncertainty of the analytical procedure. At 4 °C, five out of 15 pesticides showed negative slopes, suggesting poor stability at this temperature over the 9 weeks. The effect is even more evident at 18 °C, where practically all pesticides showed evidence of instability over the same period. Therefore, the spiked freeze-dried material A has to be stored under frozen conditions (−20 to −70 °C). This information is important for the preparation of candidate CRMs for pesticides in cucumber following this design.

**Table 5:** Stability study results for freeze-dried material A

Analyte	Slope (%/week)			Significance of slope (99 % confidence level)		
	−20 °C	4 °C	18 °C	−20 °C	4 °C	18 °C
Acetamiprid	0.129	−0.136	−0.671	No	No	Yes
Azoxystrobin	0.446	0.121	−0.581	Yes	No	Yes
Carbendazim	−0.095	−0.567	−1.192	No	Yes	Yes
Chlorpyrifos	0.109	0.051	−0.425	No	No	Yes
Cypermethrin	−0.874	−5.267	−10.391	No	Yes	Yes
Diazinon	0.120	−0.027	−0.524	No	No	Yes
α + β Endosulfan	−0.887	−0.185	−0.192	Yes	No	No
Fenitrothion	−9.423	−11.025	−32.384	Yes	Yes	Yes
Imazalil	−0.060	−1.034	−1.634	No	Yes	Yes
Imidacloprid	0.157	0.020	−0.296	No	No	Yes
Iprodione	−1.197	−2.729	−3.613	No	No	No
Malathion + malaoxon	0.041	−0.063	−0.835	No	No	Yes
Methomyl	−0.254	−0.123	−0.989	No	No	Yes
Tebuconazole	0.090	−0.371	−0.956	No	Yes	Yes
Thiabendazole	0.103	−0.211	−0.804	No	No	Yes

### Conclusions and outlook

Two complementary analytical procedures were successfully developed and validated and were deemed to be fit-for-purpose. LC–MS/MS- and GC–MS/MS-based procedures were applied during this feasibility study for the production of a CRM. The evaluation of the three different processing methods for the cucumber materials suggested that sufficient homogeneity could be achieved by all the approaches assessed. For the freeze-dried spiked slurry small losses of some pesticides were attributed to the freeze-drying process itself. Reconstitution of the cucumber powder with water was found to be satisfactory, allowing small sample sizes to be utilized. Furthermore, having low water content in the sample stored at low temperatures prevented biological activity of the material and thus minimized the degradation of the pesticides. The stability study of the spiked freeze-dried cucumber powder demonstrated that no significant instability for 13 of the 15 pesticides occurred at a storage temperature of −20 °C, with the exception of endosulfan and fenitrothion. At temperatures above −20 °C the stability of the pesticides was compromised by an observable degradation over a short period of time. The findings of this study will provide useful knowledge for the future production of a matrix reference material for the selected pesticides in a high water content vegetable.

## Electronic supplementary material

Below is the link to the electronic supplementary material.

ESM 1 (PDF 32 kb)
