# Supplementary material for: Development of a new cucumber reference material for pesticide residue analysis: feasibility study for material processing, homogeneity and stability assessment
Source: Anal Bioanal Chem. 2015 Jan 28;407(11):3083–91. doi: 10.1007/s00216-015-8476-x (PMC4383829; doi:10.1007/s00216-015-8476-x)
Supplement: Supplementary file 1 — (PDF 32 kb) [file 216_2015_8476_MOESM1_ESM.pdf]

Analytical and Bioanalytical Chemistry

Electronic Supplementary Material

Development of a new cucumber reference material for pesticide residue analysis: Feasibility study for material processing, homogeneity and stability assessment

Susana Grimalt, Stefan Harbeck, Penka Shegunova, John Seghers, Berit Sejerøe-Olsen, Håkan Emteborg, Marta Dabrio

Annex I: UPLC-MS/MS: MS/MS transitions

Table S1 Tandem mass spectrometry parameters optimized at triple quadrupole instrument Xevo-TQS for native pesticides. The transitions are defined as Q for quantification and q_1 and q_2 for confirmation purpose

Analyte	Rt (min)	MS/MS transitions	Cone (V)	Collision (eV)
Acetamiprid	4.36	223 > 90 (q_2)	10	30
		223 > 126 (Q)	10	25
		223 > 128 (q_1)	10	20
Azoxystrobin	6.01	404 > 172 (q_2)	15	55
		404 > 329 (Q)	15	30
		404 > 344 (q_1)	15	25
Carbendazim	4.57	192 > 105 (q_2)	30	35
		192 > 132 (q_1)	30	30
		192 > 160 (Q)	30	15
Chlorpyriphos	7.23	349.9 > 124.9 (q_2)	40	20
		349.9 > 197.9 (Q)	40	20
		351.9 > 199.9 (q_1)	40	20
Diazinon	6.69	305 > 97 (q_2)	5	35
		305 > 153 (q_1)	5	20
		305 > 169 (Q)	5	20
Imazalil	6.66	297 > 69 (q_1)	10	20
		297 > 159 (Q)	10	25
		299 > 161 (q_2)	10	30
Imidacloprid	4.16	256 > 84 (q_2)	25	15
		256 > 175 (q_1)	25	20
		256 > 209 (Q)	25	15
Malathion	6.25	331 > 79 (q_2)	5	40
		331 > 127 (Q)	5	10
		331 > 285 (q_1)	5	5
Malaoxon	5.32	315 > 99 (Q)	5	20
		315 >127 (q_1)	5	15
		315 >143 (q_2)	5	10
Methomyl	3.57	163 > 88 (Q)	5	10
		163 > 106 (q_1)	5	10

		163 >122 (q_2)	5	5
Tebuconazole	6.65	308 > 70 (Q)	5	30
		308 >125 (q_2)	5	35
		310 > 70 (q_1)	5	20
Thiabendazole	4.94	202 > 65 (q_2)	10	40
		202 > 131 (q_1)	10	30
		202 > 175 (Q)	10	25
Thiodicarb	5.54	355 > 88 (Q)	10	15
		355 > 108 (q_1)	10	15
		355 > 163 (q_2)	10	5

Table S2 Tandem mass spectrometry parameters optimized at triple quadrupole instrument Xevo-TQS for isotopic labelled pesticides

**Analyte**	**Rt (min)**	**MS/MS transitions**	**Cone (V)**	**Collision (eV)**
Acetamiprid d_3	4.36	226 > 126	10	20
Azoxystrobin d_4	6.01	408 > 333	15	35
Carbendazim d_4	4.57	196 > 164	30	20
Chlorpyriphos d_{10}	7.23	360 > 131	40	20
Diazinon d_{10}	6.69	315 > 170	5	20
Fenitrothion d_6	6.27	284 > 131	15	25
Imazalil d_5	6.66	302 > 159	15	20
Imidacloprid d_4	4.16	260 > 213	25	15
Malathion d_{10}	6.25	341 > 132	5	10
Malaoxon d_6	5.32	322 > 100	25	25
Methomyl d_3	3.57	166 > 88	5	10
Tebuconazole d_6	6.65	314 > 72	5	20
Thiabendazole d_6	4.94	208 > 136	10	30
Thiodicarb d_6	5.54	361 > 88	15	15

Annex II: GC-MS/MS: MS/MS transitions

Table S3 Tandem mass spectrometry parameters optimized at triple quadrupole instrument TSQ Quantum XLS for native pesticides. The transitions are defined as Q for quantification and q_1 and q_2 for confirmation purpose

Analyte	Rt window (min)	Collision Energy (V)	Transition
Azoxystrobin	17.3-18.3	25	344 > 329 (Q)
		25	388 > 300 (q_1)
		20	388 > 345 (q_2)
Cypermethrin	15.7-16.5	30	181 > 152 (Q)
		30	181 > 153 (q_1)
		30	163 > 91 (q_2)
Diazinon	9.5-10.2	15	304 > 179 (q_1)
		15	304 > 180 (Q)
		25	179 > 137 (q_2)
α + β Endosulfan	12.0-13.4	20	339 > 195 (Q)
		20	339 > 197 (q_1)
		25	241 > 171 (q_2)
		25	241 > 206 (q_3)
Fenitrothion	10.7-11.4	10	277 > 260 (Q)
		20	277 > 109 (q_1)
		15	260 > 125 (q_2)
Iprodione	13.7-14.4	20	314 > 245 (Q)
		25	316 > 247 (q_1)
Malathion	10.7-11.4	15	173 > 99 (Q)
		15	173 > 117 (q_1)
		15	127 > 99 (q_2)

Table S4 Tandem mass spectrometry parameters optimized at triple quadrupole instrument TSQ Quantum XLS for isotopic labelled pesticides

Analyte	Rt window (min)	Collision Energy (V)	Transition
Azoxystrobin d_4	17.3-18.3	30	348 > 332
Cypermethrin $^{13}C_6$	15.7-16.5	30	187 > 158
Diazinon d_{10}	9.5-10.2	25	183 > 123
α+ β Endosulfan $^{13}C_9$	12.0-13.4	15	348 > 132
Fenitrothion d_6	10.7-11.4	20	266 > 131
Iprodione d_7	13.7-14.4	25	320 > 192
Malathion d_{10}	10.7-11.4	15	183 > 100
